# Hepatitis E virus seroprevalence and determinants in various study populations in the Netherlands

**DOI:** 10.1371/journal.pone.0208522

**Published:** 2018-12-17

**Authors:** C. J. Alberts, M. F. Schim van der Loeff, S. Sadik, F. R. Zuure, E. J. A. J. Beune, M. Prins, M. B. Snijder, S. M. Bruisten

**Affiliations:** 1 Department of Infectious Diseases, Public Health Service Amsterdam (GGD), Amsterdam, the Netherlands; 2 Division of Infectious Diseases, Department of Pediatrics, Stanford University School of Medicine, Stanford, California, United States of America; 3 Amsterdam Infection and Immunity Institute, Academic Medical Center, University of Amsterdam, Amsterdam, the Netherlands; 4 Department of Public Health, Academic Medical Centre (AMC), University of Amsterdam, Amsterdam, the Netherlands; University of Cincinnati College of Medicine, UNITED STATES

## Abstract

**Background:**

The epidemiology of hepatitis E virus (HEV) is not fully understood. In this study, we assessed putative risk factors for HEV seropositivity in various study populations in the Netherlands.

**Methods:**

Data and samples from five different study populations were analysed: (A) blood donors (n = 5,239), (B) adults reporting a vegetarian life style since the age of 12 years (n = 231), (C) residents of Amsterdam, the Netherlands, with different ethnic backgrounds (n = 1,198), (D) men who have sex with men (MSM) (HIV positive and HIV negative) (n = 197), and (E) persons who use drugs (PWUD) (HIV positive and HIV negative) (n = 200). Anti-HEV immunoglobulin M (IgM) and immunoglobulin G (IgG) testing was performed using ELISA test (Wantai).

**Results:**

HEV IgM seroprevalence was low across all study populations (<1% to 8%). The age and gender-adjusted HEV IgG seroprevalence was 24% among blood donors (reference group) and 9% among the vegetarian group (adjusted Relative Risk [aRR]:0.36, 95%CI:0.23–0.57). Among participants of different ethnic backgrounds, the adjusted HEV IgG seroprevalence was 16% among participants with a Dutch origin (aRR:0.64, 95%CI:0.40–1.02), 2% among South-Asian Surinamese (aRR:0.07, 95%CI:0.02–0.29), 3% among African Surinamese (aRR:0.11, 95%CI:0.04–0.34), 34% among Ghanaian (aRR:1.53, 95%CI:1.15–2.03), 19% among Moroccan (aRR:0.75, 95%CI:0.49–1.14), and 5% among Turkish (aRR:0.18, 95%CI:0.08–0.44) origin participants. First generation Moroccans had a higher risk for being IgG HEV seropositive compared to second generation Moroccan migrants. The statistical power to perform these analyses in the other ethnic groups was too low. In the MSM group the IgG HEV seroprevalence was 24% (aRR:0.99, 95%CI:0.76–1.29), and among PWUD it was 28% (aRR:1.19, 95%CI:0.90–1.58). The number of sexual partners in the preceding six months was not significantly associated with IgG HEV seropositivity in MSM. The association between HIV status and HEV seropositivity was significant in PWUD, yet absent in MSM. HIV viral load and CD4 cell count were not associated with HEV seropositivity in HIV positive MSM and PWUD.

**Conclusions:**

Vegetarians were significantly less often HEV seropositive. Ethnic origin influenced the risk for being IgG HEV seropositive. MSM and PWUD were not at higher risk for being IgG HEV seropositive than blood donors.

## Introduction

The epidemiology of Hepatitis E Virus (HEV), a single-stranded non-enveloped RNA virus, is not fully understood [1]. There are four known HEV genotypes, each with different routes of transmission: genotype 1 (waterborne, human to human, probably zoonotic), genotype 2 (human to human), and genotypes 3 and 4 (zoonotic, consumption of raw or undercooked animal meat, and environmental [shellfish], and blood transfusion) [2]. Genotype 1 is most often responsible for HEV cases in Asia and Africa, genotype 2 is most often found in Mexico and Africa, genotype 3 is spread heterogeneously around the globe, but mostly found in Europe and the USA, while genotype 4 is also found worldwide, yet mostly in Southeast Asia [3,4].

Infection with HEV is currently not perceived as a threatening condition among healthy individuals and is found to clear spontaneously in most cases [2]. In some cases an acute infection can be life threatening, for example in pregnant women infected with HEV genotype 1, in organ transplant recipients, and in other immunosuppressed individuals [2,3]. As these groups are at higher risk to receive a blood transfusion, an important open question is whether blood donors should be screened for HEV infection [1]. In the Netherlands, a country categorized as low-endemic for HEV, the seroprevalence among blood donors has recently been estimated to be approximately 27% (95%CI: 26–28) [5]. Risk-behaviour based donor selection is one of the cornerstones of a safe blood supply in most western countries [6]. In relation to the discussion whether blood donors should be screened for HEV infection, it is of key importance to identify sub-populations at elevated risk for HEV infection to take the necessary precautions when risk-behaviour based donor selection would be implemented for HEV.

It was long thought that HEV in western societies was restricted to travellers returning from endemic regions. However, evidence is accumulating that HEV, specifically genotypes 3 and 4, may be locally acquired via exposure to, for example rats, wild boar, and rabbits, and likely most importantly via pigs (zoonotic or by consumption) [3,7]. Other possible routes of transmission that have not been investigated in detail are sexual transmission, a known transmission route for hepatitis B virus (HBV) [8,9] and hepatitis C virus (HCV) [10] among men who have sex with men (MSM), or by sharing (injecting) drug equipment. Non-western migrant populations may be potential risk groups for acquiring an HEV infection, as many are born in or regularly travel to their country of birth, which often has HEV endemic areas.

We identified the following study populations: (A) blood donors from the Netherlands (acting as reference population, data previously published (5)), (B) adults reporting a vegetarian life style since the age of 12 years (vegan/vegetarian/flexitarian), (C) participants having different ethnic backgrounds (i.e. of South-Asian Surinamese, African Surinamese, Ghanaian, Moroccan, Turkish, or Dutch origin), (D) MSM (HIV positive and HIV negative), and (E) persons who use drugs (PWUD) (HIV positive and HIV negative). In these study populations we aimed to identify those populations at higher (or lower) risk for being anti-HEV immunoglobulin M (IgM) and anti-HEV immunoglobulin G (IgG) seropositive compared to blood donors, and to assess putative risk factors for being HEV seropositive.

## Methods

### Study populations

The following study populations were analysed (S1 File):

#### Population A: Blood donors from the Netherlands

Data were provided by the authors from an HEV seroprevalence study among Dutch blood donors, as published by Slot *et al*. [5]. In short, on two days in March 2011 blood donors from the Dutch blood collection centres were approached for additional testing on HEV. In total 5,239 blood donors consented to participate. No additional information was collected apart from the information routinely retrieved (gender, year of birth, and geographic region).

#### Population B: Participants with a vegetarian life style with low or no meat intake

Participants aged ≥18 years were recruited from November 2014 through November 2015 in and near Amsterdam, the Netherlands, and were included if they reported eating meat, fish, and shellfish ≤1 time per week since the age of 12 years. This group contained participants with a vegan, vegetarian and flexitarian diet. A person was considered flexitarian if he/she ate mainly vegetarian food but occasionally also meat. Recruitment was done via online adds and flyers that were distributed via social media, vegan fairs, and word of mouth advertisement. Participants were invited to visit the Public Health Service of Amsterdam (the Netherlands) and provided blood samples and completed a questionnaire on their socio-demographic characteristics and on their food frequency behaviour in the past month (S2 File). In total, 236 participants were recruited. Four participants did not complete the questionnaire or did not provide a blood sample and one participant was excluded because she reported eating meat, fish, or shellfish more than once a week, resulting in 231 included participants. For brevity, in the remainder of this manuscript we will refer to this group as ‘vegetarians’. The study was approved by the AMC Ethical Review Board (protocol number NL50095.018.14). All participants provided written informed consent.

#### Population C: Adults of different ethnic backgrounds from the general population

Participants were selected from the multi-ethnic Healthy Life In an Urban Setting (HELIUS) study, which included participants (18–70 years) of various ethnic backgrounds (i.e. of Dutch, South-Asian Surinamese, African Surinamese, Ghanaian, Moroccan, or Turkish origin) [11,12]. Participants were randomly selected, by ethnicity, through the municipality register of Amsterdam. Ethnicity was based on the country of birth of the participant and the country of birth of both parents. A participant was considered of non-Dutch ethnic origin if he or she was (I) born outside the Netherlands and had at least one parent who was born outside the Netherlands (first generation) or (II) the participant was born in the Netherlands and both her/his parents were born outside the Netherlands (second generation) [11,13]. Participants provided blood samples and completed an extensive questionnaire on socio-demographic characteristics [11,12]. All participants, except Ghanaians, were offered an additional food frequency questionnaire tailored towards their eating habits in the past month [14]. The food frequency questionnaire used for the Ghanaian population was a questionnaire used in the RODAM (Research on Obesity and Diabetes among African Migrants) study [15–17], which is embedded in the HELIUS study, and they answered questions regarding their food intake in the past 12 months.

Our study sample was selected from 13,316 participants that were included into HELIUS between January 2011 and June 2014. For the current study, we excluded participants if they did not give permission to store biological samples (n = 883), if not enough blood was available to perform laboratory analyses (n = 675), if ethnicity was ‘other’ or ‘unknown’ (n = 26), if being of Surinamese origin other than South-Asian Surinamese or African Surinamese (n = 254), and if aged ≥45years (n = 6,234). After these exclusions, 5,244 participants were eligible of whom 1,200 participants were randomly selected for this study stratified by ethnicity (6 ethnic groups, n = 200 each). Of the 1,200 selected participants, one participant was excluded because no serum sample was found, and one other participant withdrew consent later on in the study, resulting in a total of 1,198 participants. The HELIUS study was approved by the AMC Ethical Review Board (protocol number:10/100; amendment10/100# 10.17.1729) and all participants provided written informed consent.

#### Population D: Amsterdam Cohort Study of men who have sex with men (MSM)

The ongoing Amsterdam Cohort Studies (ACS) on HIV among MSM aged ≥18 years was initiated in 1984. ACS was started to investigate the epidemiology, risk factors, and the natural history of HIV and sexual risk behaviour pattern among MSM [18,19], but the focus has now broadened to study other blood or sexually transmitted infections besides HIV. The AMC Ethical Review Board approved this study (most recent approval: 2007, file number 07/182 # 07.17.1127). In total 100 HIV negative and 100 HIV positive MSM aged ≥18 years who provided a blood sample between 1986 and 2012 were randomly selected. Three participants were excluded because they were born as a woman, resulting in 197 included MSM. Socio-demographic characteristics, number of sexual partners in the past six months, HIV status, CD4 cell count and HIV viral load were available (S3 File).

#### Population E: Amsterdam Cohort Study on persons who use drugs (PWUD)

The Amsterdam Cohort Studies on HIV among PWUD was initiated in December 1985 [20]. This cohort was initially started to study the epidemiology, risk factors and the natural history of HIV. The AMC Ethical Review Board approved this study (most recent approval: 2009, file number 09/040 # 09.17.0723). In total 100 HIV negative and 100 HIV positive PWUDs who provided a blood sample between 1992 and 2011 were randomly selected from the ACS. Socio-demographic characteristics, injecting drug use (never/preceding six months/ever-but not in the preceding six months), HIV status, CD4 cell count and HIV viral load were collected within ACS (S3 File).

### Laboratory analyses

Ethylene-diamine-tetra-acetic acid (EDTA) blood samples from the participants from study populations A, B and C, and serum samples from populations D and E were tested for antibodies against HEV (anti-HEV IgM, anti-HEV IgG) by means of an enzyme immunoassay according to instructions of the manufacturer (Wantai Biological Pharmacy Enterprise Co., Ltd, Beijing, China).

### Statistical analyses

Demographic characteristics (age and gender) were compared between the study populations using Pearson’s Chi-squared test for categorical variables and the Kruskal-Wallis test for continuous variables. *P*-values were obtained by comparing the study populations B through E to those of the blood donor population (study population A). We used the blood donor population as a reference population to investigate whether specific groups were at higher or lower risk for HEV; in this way we evaluated whether blood donation protocols should be adapted.

Anti-HEV IgM and IgG seroprevalences are presented crude, and after correction for gender and age using average marginal predicted probabilities [21]. Anti-HEV IgG seropositivity was compared between study populations by estimating the relative risk (RR) using Poisson regression analyses using log as link function and robust error variance [22]. We assessed the association between anti-HEV IgG seropositivity and study population while adjusting for age and gender. Poisson regression analyses using log as link function was used to answer the outlined research questions. We estimated the probability of anti-HEV IgG seropositivity as a function of age in years and as a function of year of birth, using restricted cubic spline standard logistic regression models with 4 knots (at 5%, 35%, 65%, and 95%). Statistical analyses were performed using Stata 14 (Stata Intercooled, College Station, TX, USA) [23]. Statistical significance was set at *p*<0.05.

## Results

### Descriptive

Data of in total 7,065 participants were analysed and 3,958 (56%) were male and 3,107 (44%) were female (S1 Table). Median age was 46 years (IQR: 35–56). Age and gender distribution of the various study populations differed significantly from that of the blood donors, with the blood donors being older and more often being male (S1 Table).

### Overall IgM and IgG HEV seroprevalence

**Fig 1:**
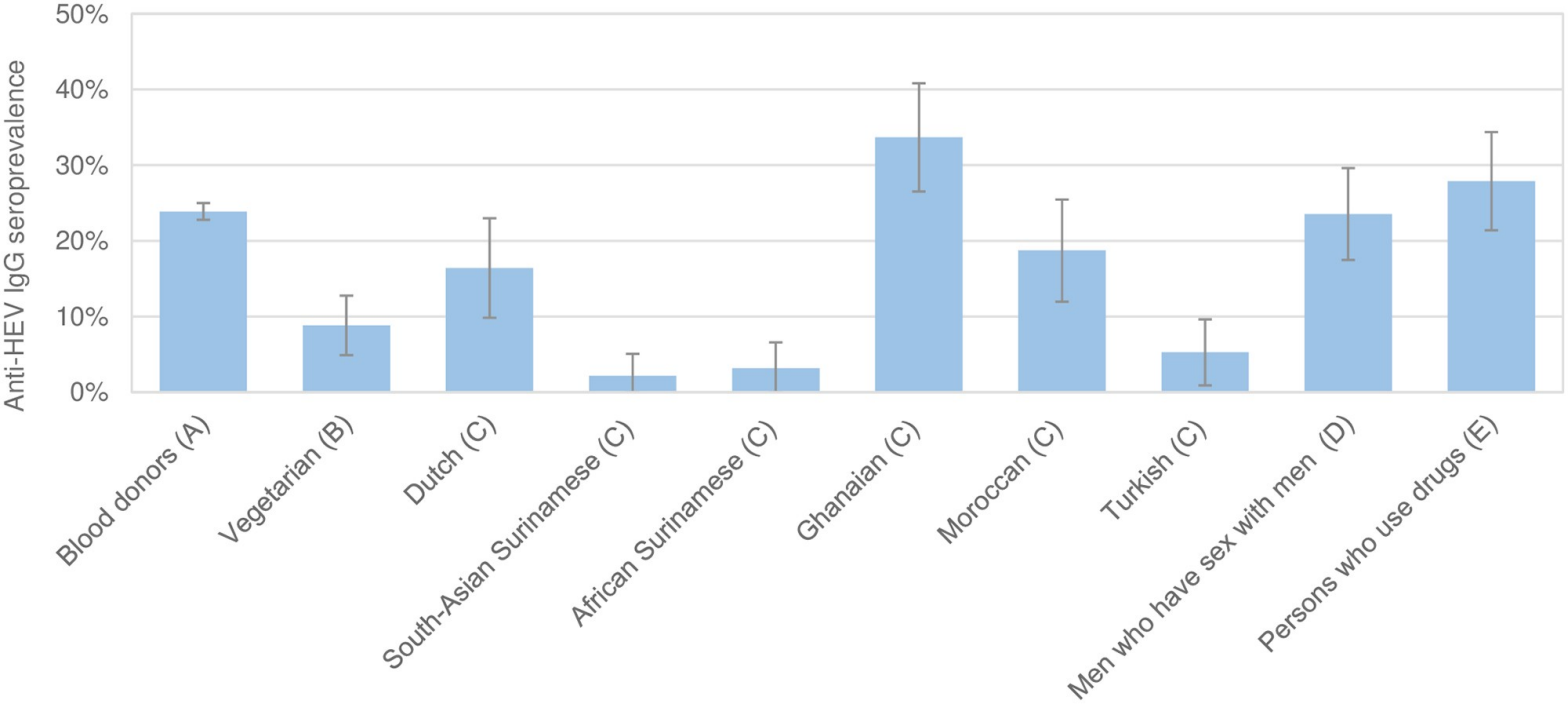
Anti-HEV IgG seroprevalence stratified by study population, corrected for age and gender. The letter presented behind the name of the group indicates the study population as described in the method section. The error bars depict the 95% confidence interval for the expected IgG HEV seroprevalence.

In Table 1 the seroprevalence of IgM HEV and IgG HEV in the various study populations is presented. The IgM HEV seroprevalence was 8% among the blood donors and 1% or lower in all other study populations. The age and gender adjusted IgG HEV seroprevalence by study population is also presented in Fig 1. After adjustment for age and gender we observed that compared to blood donors (study population A), the vegetarians (study population B), South-Asian Surinamese, African Surinamese and Turkish (study population C) had a significantly lower IgG HEV seroprevalence (Table 1 and Fig 1). HELIUS participants with a Dutch or Moroccan origin (study population C), MSM (study population D) and PWUD (study population E) had similar IgG HEV seroprevalences compared to blood donors. HELIUS participants with a Ghanaian background (study population C) had a significantly higher risk to be IgG HEV seropositive compared to the blood donors (study population A).

**Table 1:** Anti-HEV immunoglobulin M (IgM) and IgG seroprevalence in various research populations from the Netherlands.

	Anti-HEV IgM seroprevalence			Anti-HEV IgG seroprevalence			Anti-HEV IgG Seroprevalence (age and gender^a^)		Anti-HEV IgG	
	n	N	%	n	N	%	%	(95%CI)	aRR^b^	95% CI
Blood donors	429	5,239	8%	1,401	5,239	27%	24%	(23%-25%)	REF	
Vegetarians^c^	1	231	0.43%	17	231	7%	9%	(5%-13%)	**0.36**	**(0.23–0.57)**
Dutch	2	200	1%	17	200	9%	16%	(10%-23%)	0.64	(0.40–1.02)
South-Asian Surinamese	0	200	0%	2	200	1%	2%	(0%-5%)	**0.07**	**(0.02–0.29)**
African Surinamese	1	199	0.50%	3	199	2%	3%	(0%-7%)	**0.11**	**(0.04–0.34)**
Ghanaian	1	199	0.50%	44	199	22%	34%	(27%-41%)	**1.53**	**(1.15–2.03)**
Moroccan	1	200	0.50%	20	200	10%	19%	(12%-25%)	0.75	(0.49–1.14)
Turkish	0	200	0%	5	200	3%	5%	(1%-10%)	**0.18**	**(0.08–0.44)**
Men who have sex with men	0	197	0%	40	197	20%	24%	(17%-30%)	0.99	(0.76–1.29)
Persons who use drugs	0	200	0%	42	200	21%	28%	(21%-34%)	1.19	(0.90–1.58)

^a^ Age and gender adjusted seroprevalence using the average marginal effect.

^b^ Association between study population and anti-HEV IgG seroprevalence using Poisson Regression analyses with log as link function after correcting for age and gender

^c^ This group consists of 71 participants indicating to be vegan, 152 participants indicating to be vegetarian and 8 participants indicating to be flexitarian. The IgM seroprevalence in these three subgroups was 0% (0/71), <1% (1/152) and 0% (0/8) respectively. The IgG seroprevalence in these three subgroups was 7% (5/71), 8% (12/152) and 0% (0/8) respectively.

Abbreviations: HEV = hepatitis E virus, immunoglobulin M = IgM, immunoglobulin G = IgG, aRR = adjusted Relative Risk, CI = Confidence Interval.

For analytic purposes age was modelled using restricted cubic splines with knots at the 5th, 35th, 65th and 95th percentile.

Percentages <1% are reported with two decimals.

### IgG HEV seroprevalence by age and by year of birth

**Fig 2:**
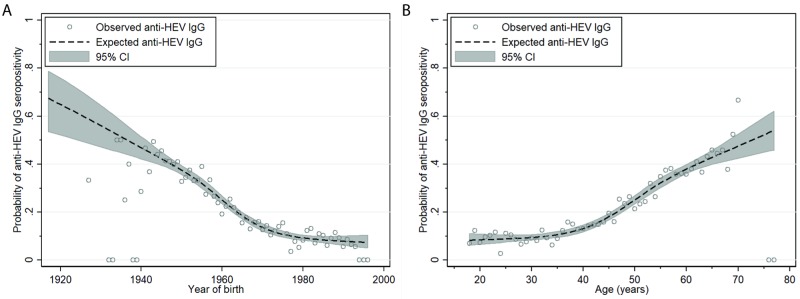
Estimated probability of IgG HEV seroprevalence by age (left, p<0.001) and by year of birth (right, p<0.001) among 7,065 participants of the total study population. Expected IgG HEV seroprevalence is derived from a 4-knot restricted cubic spline standard logistic regression model using default knot values (dashed line). The grey shading depicts the 95% confidence interval for expected IgG HEV seroprevalence. Dots represent the observed seroprevalence of IgG HEV per age-year and per birth-year.

The estimated probability of IgG HEV seropositivity increased with age (*p*<0.001) and decreased accordingly with year of birth (*p*<0.001) (Fig 2).

### IgG HEV seroprevalence by ethnicity and migration status

In Table 2 the IgG HEV seroprevalence of the non-Dutch participants is compared to the Dutch group within the HELIUS study (study population C). After adjustment for age and gender we found that South-Asian Surinamese, African Surinamese and Turkish participants had a significantly lower IgG HEV seroprevalence compared to the Dutch participants. Ghanaian participants had a significantly higher IgG HEV seroprevalence, while Moroccan participants had an IgG HEV seroprevalence similar to that of the Dutch participants. In Table 2 the IgG HEV seroprevalence is also stratified by migration generation status within each ethnicity. Because of very low numbers within the first or second generation group for most ethnic groups, we only made a statistical comparison of IgG HEV seroprevalence between first and second generation migrants for Moroccan participants. Participants who were born in the Netherlands and of whom both parents were born in Morocco (second generation) had a significantly lower IgG HEV seroprevalence than participants born in Morocco with at least one other parent born in Morocco (first generation).

**Table 2:** Anti-HEV immunoglobulin G (IgG) seroprevalence by migration status among participants with different ethnic backgrounds (i.e. of South-Asian Surinamese, African Surinamese, Ghanaian, Moroccan, Turkish, or Dutch origin).

		Anti-HEV IgG seroprevalence						
		n	N	%	RR	95% CI	aRR ^a^	95% CI
Dutch		17	200	9%	REF		REF	
South-Asian Surinamese		2	200	1%	**0.12**	**(0.03–0.50)**	**0.12**	**(0.03–0.50)**
African Surinamese		3	199	2%	**0.18**	**(0.05–0.60)**	**0.16**	**(0.05–0.54)**
Ghanaian		44	199	22%	**2.60**	**(1.54–4.39)**	**2.06**	**(1.23–3.45)**
Moroccan		20	200	10%	1.18	(0.64–2.18)	1.15	(0.63–2.11)
Turkish		5	200	3%	**0.29**	**(0.11–0.78)**	**0.28**	**(0.11–0.75)**
South-Asian Surinamese	First generation	1	102	1%	N.E. ^b^		N.E. ^b^	
	Second generation	1	98	1%				
African Surinamese	First generation	1	120	1%	N.E. ^b^		N.E. ^b^	
	Second generation	2	79	3%				
Ghanaian	First generation	44	197	22%	N.E. ^b^		N.E. ^b^	
	Second generation	0	2	0%				
Moroccan	First generation	18	114	16%	REF		REF	
	Second generation	2	86	2%	0.15	(0.03–0.62)	0.20	(0.04–0.91)
Turkish	First generation	5	119	4%	N.E. ^b^		N.E. ^b^	
	Second generation	0	81	0%				

^a^ Adjusted relative risk after correcting for age and gender.

^b^ Not estimated because numbers in some groups were ≤1.

Abbreviations: HEV = hepatitis E virus, immunoglobulin G = IgG, N.E. = Not estimated.

### Association between meat and shellfish intake and IgG HEV seropositivity

In Table 3 the association between both meat and shellfish intake with IgG HEV seropositivity is presented for participants with a vegetarian life style (study population B) and HELIUS participants from different ethnic backgrounds (study population C). Among the HELIUS participants we observed that those with a vegetarian diet had a lower IgG HEV seroprevalence compared to those not having a vegetarian diet (yet this was not statistically significant). We did not observe differences between vegetarians and flexitarians within study population B. When exploring the association of IgG HEV with individual meat consumption (analysed separately for beef, poultry, lamb, minced meat, deli meat, and sausages) and shell-fish consumption, no clear associations of a particular food type with IgG HEV was found consistently across these study populations (Table 3).

**Table 3:** Meat and shellfish intake in the preceding 1–12 months by IgG HEV among various study populations from the Netherlands.

	Study population B			Study population C																	
	Vegetarians^a^			Dutch			South-Asian Surinamese			African Surinamese			Ghanaian			Moroccan			Turkish		
	(N = 231)			(N = 200)			(N = 200)			(N = 199)			(N = 199)			(N = 200)			(N = 200)		
	n	N	%	n	N	%	n	N	%	n	N	%	n	N	%	n	N	%	n	N	%
**Vegetarian diet**																					
		*p* = 0.417			*p* = 0.349			*p* = 0.696			*p* = 0.756			*p* = 0.288			*p* = 0.369			*p* = 0.641	
Vegetarian diet	17	223	8%	0	9	0%	0	14	0%	0	6	0%	0	4	0%	0	7	0%	0	8	0%
No vegetarian diet	0	8	0%	17	191	9%	2	185	1%	3	190	2%	38	172	22%	20	193	10%	5	189	3%
**Meat consumption**																					
Beef																					
		*p* = 0.570			*p* = 0.138			*p* = 0.311			*p* = 0.184			*p* = 0.416			*p* = 0.801			*p* = 0.928	
No	17	227	7%	1	39	3%	2	132	2%	0	72	0%	2	15	13%	6	65	9%	2	75	3%
Yes	0	4	0%	16	161	10%	0	67	0%	3	124	2%	36	161	22%	14	135	10%	3	122	2%
Pork																					
		*p* = 0.570			*p* = 0.320			*p* = 0.362			*p* = 0.659			*p* = 0.892			*N*.*A*.			*N*.*A*.	
No	17	227	7%	4	69	6%	2	141	1%	1	90	1%	26	122	21%	20	200	10%	5	197	3%
Yes	0	4	0%	13	131	10%	0	58	0%	2	106	2%	12	54	22%						
Poultry																					
		*p* = 0.570			*p* = 0.738			*p* = 0.644			*p* = 0.702			*p* = 0.298			*p* = 0.712			*p* = 0.531	
No	17	227	7%	2	29	7%	0	19	0%	0	9	0%	1	11	9%	1	14	7%	0	14	0%
Yes	0	4	0%	15	171	9%	2	180	1%	3	187	2%	37	165	22%	19	186	10%	5	183	3%
Lamb																					
		*p* = 0.623			*N*.*A*.			*p* = 0.110			*p* = 0.214			*p* = 0.471			*p* = 0.541			*p* = 0.878	
No	17	228	7%	17	200	9%	2	88	2%	1	131	1%	8	45	18%	5	62	8%	1	45	2%
Yes	0	3	0%				0	111	0%	2	65	3%	30	131	23%	15	138	11%	4	152	3%
Minced meat																					
		*p* = 0.524			*p* = 0.303			*p* = 0.972			*p* = 0.294			*p* = 0.508			*p* = 0.057			*p* = 0.377	
No	17	226	8%	4	30	13%	1	102	1%	0	52	0%	16	66	24%	0	28	0%	0	26	0%
Yes	0	5	0%	13	170	8%	1	97	1%	3	144	2%	22	110	20%	20	172	12%	5	171	3%
Deli meat																					
		*p* = 0.524			*p* = 0.362			*p* = 0.328			*p* = 0.369			*p* = 0.308			*p* = 0.636			*p* = 0.672	
No	17	226	8%	1	26	4%	0	64	0%	0	41	0%	22	89	25%	8	90	9%	1	56	2%
Yes	0	5	0%	16	174	9%	2	135	1%	3	155	2%	16	87	18%	12	110	11%	4	141	3%
Sausage																					
		*p* = 0.570			*p* = 0.806			*N*.*A*.			*N*.*A*.			*p* = 0.914			*N*.*A*.			*N*.*A*.	
No	17	227	7%	7	88	8%	2	199	1%	3	196	2%	20	94	21%	20	200	10%	5	197	3%
Yes	0	4	0%	10	112	9%							18	82	22%						
**Shellfish consumption**																					
		*p* = 0.945			*p* = 0.476			*p* = 0.788			*p* = 0.585			*p* = 0.715			*p* = 0.832			*p* = 0.667	
No	15	205	7%	8	78	10%	1	81	1%	1	96	1%	7	29	24%	5	54	9%	4	140	3%
Yes	2	26	8%	9	122	7%	1	118	1%	2	100	2%	31	147	21%	15	146	10%	1	56	2%

^a^ This group consists of 71 participants indicating to be vegan, 152 participants indicating to be vegetarian and 8 participants indicating to be flexitarian.

Data were missing for vegetarian status (n = 30), beef consumption (n = 30), pork consumption (n = 30), poultry consumption (n = 30), lamb consumption (n = 30), minced meat consumption (n = 30), deli consumption (n = 30), sausage consumption (n = 30), shellfish consumption (n = 31).

Abbreviations: HEV = hepatitis E virus, immunoglobulin G = IgG.

### IgG HEV seroprevalence by number of sexual partners among MSM

The number of sexual partners in the preceding six months was not significantly associated with IgG HEV seropositivity among MSM in both crude and multivariable analyses (Table 4).

**Table 4:** Anti-HEV immunoglobulin G (IgG) seroprevalence among men who have sex with men (n = 197).

	Anti-HEV IgG			Anti-HEV IgG		Anti-HEV IgG	
	seroprevalence						
	n	N	%	RR	95% CI	aRR^a^	95% CI
**Number of sexual partners in the preceding six months**							
0–1	15	76	20%	REF		REF	
2–9	10	42	24%	1.21	(0.59–2.45)	1.02	(0.55–1.89)
≥10	8	40	20%	1.01	(0.47–2.19)	0.92	(0.46–1.83)
**HIV status**							
HIV negative	20	99	20%	REF		REF	
HIV positive	20	98	20%	1.01	(0.58–1.76)	1.17	(0.67–2.06)
**HIV positive men who have sex with men (n = 98)**							
**Number of sexual partners in the preceding six months**^c^							
0–1	8	33	24%	REF		NE^b^	
2–9	3	16	19%	0.77	(0.23–2.55)		
≥10	3	13	23%	0.95	(0.30–3.07)		
**HIV viral load (copies/ml)**							
≤10,000	5	33	15%	REF		NEb	
>10,000	4	24	17%	1.10	(0.33–3.71)		
**CD4 cell count (cells/ul)**							
<500	13	46	28%	REF		NEb	
≥500	4	35	11%	0.40	(0.14–1.14)		

^a^ Adjusted relative risk (aRR) after correcting for age, number of sexual partners and HIV status. Multivariable model contains 158 participants.

^b^ RR not estimated due to low statistical power.

^c^ Data were missing for number of sexual partners in the preceding six months (n = 36), CD4 cell count (n = 17) and HIV viral load (n = 41).

Abbreviations: HEV = hepatitis E virus, immunoglobulin G = IgG, aRR = adjusted Relative Risk, CI = Confidence Interval, HIV = human immunodeficiency virus, NE = Not Estimated.

### IgG HEV seroprevalence by injecting drug behaviour

Among PWUD reporting to never have injected drugs had a non-significant lower risk to be IgG HEV seropositive compared to those reporting to inject drugs in the preceding six months. Participants who reported ever having injected drugs (but not in the preceding six months) had a similar risk to be IgG HEV seropositive compared to participants reporting to have injected drugs in the preceding six months (Table 5).

**Table 5:** Anti-HEV immunoglobulin G seroprevalence among persons who use drugs (n = 200).

	Anti-HEV IgG			Anti-HEV IgG		Anti-HEV IgG	
	seroprevalence						
	n	N	%	RR	95% CI	aRR^a^	95% CI
**Injecting drug behaviour**							
Never	3	28	11%	0.48	(0.15–1.51)	0.46	(0.15–1.43)
Preceding 6 months	20	90	22%	REF		REF	
Ever-but not in preceding 6 months	17	73	23%	1.05	(0.59–1.85)	1.05	(0.60–1.84)
**HIV status**							
HIV negative	26	100	26%	REF		REF	
HIV positive	16	100	16%	0.62	(0.35–1.08)	**0.53**	**(0.31–0.93)**
**HIV positive drug users (n = 100)**							
**Injecting drug behaviour**							
Never	0	8	0%	N.A.		NE^b^	
Preceding 6 months	6	47	13%	REF		REF	
Ever-but not in preceding 6 months	9	42	21%	1.68	(0.65–4.34)	1.65	(0.50–5.48)
**HIV viral load (copies/ml)**							
≤10,000	10	44	23%	REF		REF	
>10,000	2	21	10%	0.42	(0.10–1.76)	0.40	(0.09–1.94)
**CD4 cell count**							
<500	13	58	22%	REF		REF	
≥500	3	39	8%	0.34	(0.10–1.13)	0.29	(0.08–1.10)

^a^ Adjusted relative risk after correcting for age, gender, injecting drug behaviour, and HIV status. The same variables were used in the model among HIV positive persons who use drugs i.e. including CD4 cell count and HIV viral load. Multivariable model among total study population contains 191 participants. Multivariable model among HIV positive individuals contains 61 participants.

^b^ RR not estimated due to low statistical power.

Data were missing for injecting drug behaviour (n = 9), CD4 cell count (n = 3), and HIV viral load (n = 35).

Abbreviations: HEV = hepatitis E virus, immunoglobulin G = IgG, aRR = adjusted Relative Risk, CI = Confidence Interval, HIV = human immunodeficiency virus, NE = Not estimated.

### Association between HIV and IgG HEV seropositivity

In both crude and multivariable analyses no association between HIV status and IgG HEV seropositivity was found among MSM (Table 4). Among persons who use drugs IgG HEV seroprevalence was non-significantly lower in HIV positive when compared to HIV negative participants in crude analyses, but was significantly lower in multivariable analyses (Table 5); HIV became significantly associated with IgG HEV seropositivity after adding injecting drug behaviour to the model. CD4 cell count and HIV viral load were not significantly associated with IgG HEV seropositivity among HIV positive PWUD or HIV positive MSM (Tables 4 and 5).

## Discussion

“With so many unknowns, controlling this silent [HEV] epidemic is a challenge” [1]. To better understand the HEV epidemiology in the Netherlands, we examined and compared HEV seroprevalence in various study populations. Few studies have compared such a wide variety of study populations from the same country head-to-head within a single study on HEV. We found IgM HEV seroprevalence to be low and varying between <1 to 8%. The IgG HEV seroprevalence, however, varied markedly between the various study populations. Compared to blood donors, HEV IgG seroprevalence was significantly lower among those with a vegetarian life style, and among South-Asian Surinamese, African Surinamese and Turkish adults from the HELIUS population, whereas it was similar in those of Dutch origin, Moroccan origin, and in MSM and PWUD, and was significantly higher among participants with a Ghanaian origin.

The antibody response within humans after an HEV infection follows a conventional course: a direct increase of IgM when symptoms start, with IgM antibodies disappearing within 3 months; and an increase of IgG reaching its highest levels soon after symptoms have started (IgG antibodies remain detectable for at least 12 years and probably longer) [3,4,24]. HEV has only a narrow viremic window (3–5 weeks) [3]. Therefore, the HEV seroprevalences reported in this study are informative for the understanding of the epidemiology of past and present HEV infections within populations. However, when screening for active cases among blood donors, PCR analysis should be performed to identify acute cases.

### Age and year of birth

In this study we found a stable IgG HEV seroprevalence up to the age of 40, which gradually increased up to the age of ~70 years. Accordingly, we found an inverse pattern with year of birth; a gradual decrease of IgG HEV seroprevalence for participants born before 1970 and a steady IgG HEV seroprevalence for those born after 1970. A similar pattern has been observed previously among blood donors in the Netherlands [25]. Age-specific IgG HEV patterns are most likely influenced by the infection pressure within a country and are therefore hard to extrapolate to other countries [7].

### Ethnicity and migration status

We found that the IgG HEV seroprevalences differed significantly between participants from different ethnic backgrounds, in agreement with previous findings in Amsterdam, the Netherlands [26]. Remarkable was the low IgG HEV seroprevalence among Surinamese participants, which may be due to dietary habits: Surinamese have a “noodle/rice dishes and white meat” dietary pattern [27] in which meat is usually fried and/or fully cooked. Among individuals with a Moroccan background, we found that those born in the Netherlands (second generation) were less often HEV seropositive compared to individuals born in Morocco (first generation). This may suggest that HEV was contracted when living in Morocco, and as this group typically does not consume pork meat (based on religion), we hypothesize that these groups contracted HEV via contaminated sources, such as water in their home country. A similar pattern was observed among Turkish participants, although due to an overall lower seroprevalence we did not have the statistical power to further analyse this. Among individuals with a Ghanaian background we could not differentiate between first and second generation migrants, as only two participants were classified as second generation migrants. Yet we found that Ghanaian participants from the HELIUS study had a significantly higher IgG HEV seroprevalence when compared to individuals with a Dutch background from the HELIUS study or to blood donors. As participants with a Ghanaian origin in this study moved fairly recently to the Netherlands (median calendar year 2000 [IQR: 1994–2005]), we hypothesize that HEV was probably contracted in Ghana and this may also be due to contaminated water and/or hygienic conditions in the home country.

### Association between meat and shellfish intake and IgG HEV seropositivity

Currently it is hypothesized that the consumption of meat is one of the main transmission routes driving the HEV epidemic in western countries, with pork and sausages being the main suspects [1]. Pigs have been identified as an important reservoir for HEV and sequence analysis on HEV retrieved from pigs showed a high homology with HEV isolated from humans [4]. In this study we show that participants reporting a vegetarian lifestyle since the age of 12 years have a significantly lower HEV seroprevalence compared to blood donors, as observed previously [28]. We observed slightly higher IgG HEV seroprevalences among those reporting eating specific types of meat, yet no clear significant associations of a particular food source (including sausage and beef) was found consistently across multiple study populations. This may be explained in various ways, most likely because the numbers were too low within individual study populations. Furthermore, IgG HEV is a marker of past infection that may have happened years ago; but as we queried participants’ food consumption over the past 1–12 months, it is hard to use these data to pin-point a specific meat type as the source of a past infection. Additionally, the way meat is prepared is an important risk factor (i.e. meat should be heated up to 71°C for at least 20 minutes to completely inactivate HEV [29]). Shellfish consumption has previously been reported to be a common risk factor for contracting HEV [30,31], yet in this study no significant associations were found. Small contained outbreaks on e.g. a cruise ship (where shellfish was identified as a risk factor) may help to identify a single source of infection [30,31], although even then it can still be hard to pinpoint one single source because of the long period between infection and appearance of clinical symptoms.

### IgG HEV seroprevalence by HIV status

Most studies investigating the relation between HIV and HEV have reported a non-significant association [32–36]. Nevertheless, in multivariable analyses among persons who use drugs we found that HIV positive individuals had a lower IgG HEV seroprevalence compared to HIV negative individuals. HIV only became significantly associated with IgG HEV seropositivity after adding injecting drug behaviour to the multivariable model. The association between HIV and HEV was absent among MSM. Literature suggests that HIV infection in itself is not a risk factor for acquiring HEV infection [35,36]. Unexplained liver enzyme elevation is common among HIV-positive individuals treated with antiretroviral drugs [37,38] and evidence is accumulating that individuals developing chronic HEV are often immunocompromised [39,40]. HEV—apart from the other well known hepatitis infections (HBV and HCV)—should therefore be recognized as a possibly important opportunistic infection occurring among HIV positive individuals. Our analyses did not show an association between CD4 and IgG HEV seropositivity among HIV positive individuals, but this may be because the CD4 cell count was relatively high (43% [35/81] and 40% [39/97] above 500 cells/ul in MSM and IDU respectively). So, as this concerns a relatively healthy HIV positive population on treatment, we assume that the immunological response to HEV was similar in HIV positive to that in HIV negative participants. The few studies investigating the role of CD4 cells showed a lower HEV (sero)positivity among participants with a low CD4 cell count, but this association was not always statistically significant in multivariable analyses [32,33].

### IgG HEV seroprevalence by injecting drug use status

Sharing injecting equipment is an important risk factor for blood-borne infections such as HIV, HBV, and HCV [41–43]. Furthermore, HEV is now officially recognized as a transfusion-transmittable infection [44,45]. With HIV and HCV being common infectious diseases among persons who use drugs, HEV may also be a common pathogen in this group. Literature on this is equivocal, with some studies showing persons who inject drug having a higher HEV seroprevalence compared to controls [46,47] yet most studies have not shown an association [48–50]. In this study we found that individuals reporting to never have injected drugs had a non-significantly lower IgG HEV seroprevalence compared to those reporting to have injected drugs in the preceding six months.

### Sexual transmission of HEV

The possibility that HEV can be sexually transmitted remains controversial [35] with some suggesting it may be [51,52] while other suggest it is not [34,49,50]. Studies suggesting that HEV is a sexually transmitted infection were based on the sole fact that they found higher HEV seroprevalences in an MSM population compared to a control group. We found no differences in IgG HEV seroprevalence among MSM compared to blood donors in the Netherlands. Furthermore, we did not find any association between recent number of sexual partners and IgG HEV seropositivity. To our knowledge this is one of the first studies looking at the association between IgG HEV seropositivity and the reported number of sexual partners. Our findings suggest that IgG HEV is not sexually transmitted.

### Limitations

An important limitation of this study is that no HEV RNA typing could be performed since all tested IgM positive samples were negative when performing RT-qPCR for HEV; this is in line with what we found in a previous study on HEV [26]. So unfortunately, we could not differentiate between the various HEV types. Genotyping could have shed light on whether the Moroccan or Ghanaian population might have contracted HEV in the Netherlands or in their home country, in case they were infected with another type than genotype 3.

Furthermore, it is important to note is that, although the laboratory tests used in all study groups was the same (Wantai), the tests for the blood donor group were done in another laboratory than for the other groups within this study. The commercially available test kit has been validated and is used in many different laboratories; therefore we do not think that this will have affected results or comparability.

### Implications of study results

The question whether blood donors should be screened for the presence of an HEV infection remains under debate. To answer this question, it is of key importance to identify whether there are specific groups that are at higher risk to contract and transmit HEV. Within this study we showed that specific migrant populations, MSM, and PWUD are not at higher risk to be HEV seropositive. The IgM HEV seroprevalence was extremely low, suggesting that the prevalence of infectious HEV individuals in these specific populations is low. Based on these data we do not recommend excluding MSM and PWUD from blood donation for the purpose of HEV transmission prevention; yet these groups may be excluded from blood donation in order to avoid transmission of other infectious diseases. Whether all blood donors should be screened for HEV remains an open question. As long as the risk of acquiring HEV from the environment remains, we support the strategy to screen blood products on HEV before using these in immune-compromised individuals [1].

## Conclusions

We confirm that the risk of becoming HEV seropositive is lower for a person who follows a vegetarian (or vegan) lifestyle [28]. Our analyses furthermore show that an ethnic minority background in general does not pose a higher risk to be IgG HEV seropositive, yet in the Ghanaian study population a higher IgG HEV seroprevalence was found which was most likely caused by their migration status i.e. being born in a country with a higher HEV infection pressure compared to the Netherlands. MSM and PWUD do not have a higher risk for being IgG HEV seropositive compared to blood donors. Our study suggests that HEV is not sexually transmitted. We hypothesize that HIV status is not a risk factor, and observed that CD4 cell count and HIV viral load are not risk factors for being HEV IgG seropositive. Studies investigating incident HEV infections and the natural history of antibodies and RNA in HIV positive people should shed light on this issue.

## Supporting information

S1 Table Baseline characteristics of 7,065 participants from different research populations, the Netherlands. (DOCX)

S1 File This database contains all the data used for analyses presented in this study. (XLS)

S2 File This is the food frequency questionnaire used for study population B in English and Dutch. (DOC)

S3 File This is the questionnaire used for study population D and E in English and Dutch. (DOCX)

## Abbreviations

95% CI: 95% confidence interval
ACS: Amsterdam cohort study
aRR: Adjusted relative risk
HAV: Hepatitis A virus
HBV: Hepatitis B virus
HCV: Hepatitis C virus
HEV: Hepatitis E virus
HIV: Human immunodeficiency virus
IDU: Injecting drug user
IgG: Immunoglobulin G
IgM: Immunoglobulin M
IQR: Interquartile range
MSM: Men who have sex with men
PWUD: Persons Who Use Drugs
