# Supplementary material for: Hepatitis E virus seroprevalence and determinants in various study populations in the Netherlands
Source: PLoS One. 2018 Dec 17;13(12):e0208522. doi: 10.1371/journal.pone.0208522 (PMC6296558; doi:10.1371/journal.pone.0208522)
Supplement: S1 Table — (DOCX) [file pone.0208522.s001.docx]

| **Supplementary table 1:** Baseline characteristics of 7,065 participants from different research populations, the Netherlands | | | | | | | | | | | | | | | | | | | | | | |
| --- | --- | --- | --- | --- | --- | --- | --- | --- | --- | --- | --- | --- | --- | --- | --- | --- | --- | --- | --- | --- | --- | --- |
|  | A | | B | | C | | | | | | | | | | | | D | | E | |  |  |
|  | Blood donors (n=5,239) | | Vegetarians (n=231) | | Dutch (n=200) | | South-Asian Surinamese (n=200) | | African Surinamese (n=199) | | Ghanaian (n=199) | | Moroccan (n=200) | | Turkish (n=200) | | Men who have sex with men (n=197) | | Persons who use drugs (n=200) | | Total (n=7,065) | |
|  |  | ***REF*** | **<0.001** | | **<0.001** | | **<0.001** | | **<0.001** | | **<0.001** | | **<0.001** | | **<0.001** | | **<0.001** | | **<0.001** | |  |  |
| **Median age (IQR)** | 51 (40-59) | | 41 (31-52) | | 33 (28-39) | | 35 (26-40) | | 36 (29-41) | | 38 (34-41) | | 34 (28-39) | | 34 (27-40) | | 41 (31-51) | | 41 (31-45) | | 46 (35-56) | |
|  |  |  |  |  |  |  |  |  |  |  |  |  |  |  |  |  |  |  |  |  |  |  |
| **Age** |  | ***REF*** | **<0.001** | | **<0.001** | | **<0.001** | | **<0.001** | | **<0.001** | | **<0.001** | | **<0.001** | | **<0.001** | | **<0.001** | |  |  |
| <20 | 62 | 1% | 1 | 0% | 2 | 1% | 7 | 4% | 7 | 4% | 0 | 0% | 7 | 4% | 8 | 4% | 0 | 0% | 0 | 0% | 94 | 1% |
| 20-24 | 307 | 6% | 23 | 10% | 22 | 11% | 31 | 16% | 18 | 9% | 0 | 0% | 23 | 12% | 26 | 13% | 3 | 2% | 6 | 3% | 459 | 6% |
| 25-29 | 285 | 5% | 28 | 12% | 47 | 24% | 42 | 21% | 29 | 15% | 17 | 9% | 34 | 17% | 27 | 14% | 34 | 17% | 27 | 14% | 570 | 8% |
| 30-34 | 252 | 5% | 25 | 11% | 43 | 22% | 19 | 10% | 37 | 19% | 35 | 18% | 43 | 22% | 43 | 22% | 34 | 17% | 35 | 18% | 566 | 8% |
| 35-39 | 332 | 6% | 30 | 13% | 37 | 19% | 43 | 22% | 39 | 20% | 71 | 36% | 44 | 22% | 44 | 22% | 21 | 11% | 25 | 13% | 686 | 10% |
| 40-44 | 532 | 10% | 33 | 14% | 49 | 25% | 58 | 29% | 69 | 35% | 76 | 38% | 49 | 25% | 52 | 26% | 29 | 15% | 53 | 27% | 1,000 | 14% |
| 45-49 | 665 | 13% | 26 | 11% | 0 | 0% | 0 | 0% | 0 | 0% | 0 | 0% | 0 | 0% | 0 | 0% | 23 | 12% | 25 | 13% | 739 | 10% |
| 50-54 | 811 | 15% | 23 | 10% | 0 | 0% | 0 | 0% | 0 | 0% | 0 | 0% | 0 | 0% | 0 | 0% | 21 | 11% | 19 | 10% | 874 | 12% |
| 55-59 | 844 | 16% | 15 | 6% | 0 | 0% | 0 | 0% | 0 | 0% | 0 | 0% | 0 | 0% | 0 | 0% | 17 | 9% | 8 | 4% | 884 | 13% |
| 60-64 | 707 | 13% | 12 | 5% | 0 | 0% | 0 | 0% | 0 | 0% | 0 | 0% | 0 | 0% | 0 | 0% | 7 | 4% | 2 | 1% | 728 | 10% |
| 65-69 | 440 | 8% | 13 | 6% | 0 | 0% | 0 | 0% | 0 | 0% | 0 | 0% | 0 | 0% | 0 | 0% | 7 | 4% | 0 | 0% | 460 | 7% |
| ≥70 | 2 | 0% | 2 | 1% | 0 | 0% | 0 | 0% | 0 | 0% | 0 | 0% | 0 | 0% | 0 | 0% | 1 | 1% | 0 | 0% | 5 | 0% |
| **Gender** | ***REF*** | | **<0.001** | | **<0.001** | | **<0.001** | | **<0.001** | | **<0.001** | | **<0.001** | | **<0.001** | | **<0.001** | | **0.485** | |  |  |
| Men | 3,119 | 60% | 47 | 20% | 81 | 41% | 89 | 45% | 64 | 32% | 69 | 35% | 75 | 38% | 93 | 47% | 197 | 100% | 124 | 62% | 3,958 | 56% |
| Women | 2,120 | 40% | 184 | 80% | 119 | 60% | 111 | 56% | 135 | 68% | 130 | 65% | 125 | 63% | 107 | 54% | 0 | 0% | 76 | 38% | 3,107 | 44% |
| **Abbreviations:** IQR=interquartile range | | | | | | | | | | | | | | | | | | | | | | |
| Age is reported in years. | | | | | | | | | | | | | | | | | | | | | | |
| No data was missing. | | | | | | | | | | | | | | | | | | | | | | |
| *p* of categorical variables are based on Pearson's Chi-squared test and *p* of continuous variables are based on the Kruskall-Wallis test; | | | | | | | | | | | | | | | | | | | | | | |
