# Supplementary material for: Hepatitis E virus seroprevalence and determinants in various study populations in the Netherlands
Source: PLoS One. 2018 Dec 17;13(12):e0208522. doi: 10.1371/journal.pone.0208522 (PMC6296558; doi:10.1371/journal.pone.0208522)
Supplement: S2 File — (DOC) [file pone.0208522.s003.doc]

# Hepatitis E Virus voedsel vragenlijst

In deze vragenlijst worden algemene vragen gesteld, en vragen over mogelijke risicofactoren voor een hepatitis E virus infectie. De vragenlijsten worden anoniem verwerkt.

Dit betekent dat uw antwoorden niet te herleiden zijn tot uw persoon.

Het invullen van de vragenlijst duurt ongeveer 10-20 minuten.

Alvast hartelijk dank voor uw medewerking!

**Datum** …./…./….……

**Hoe vaak at u sinds uw 12^e^ jaar of de afgelopen 10 jaar vlees, vis of schaaldieren?**

- Nooit
- 1 keer of minder per week
- Vaker **🡪** **Einde van de vragenlijst (geen deelname mogelijk).**

Sticker

**1. Wat zijn de 4 cijfers uw postcode?** 🞎🞎🞎🞎

**2. Bent u een man of een vrouw?**

- Man
- Vrouw

**3. Wat is uw geboortedatum?**

dag maand jaar

4**. Wat is de hoogste opleiding die u heeft gevolgd of momenteel volgt?**

- Geen schoolopleiding
- Lagere school/ basisschool
- Middelbaar Algemeen Onderwijs (MAVO, MULO, ULO)
- Voortgezet Algemeen Onderwijs (HAO, VWO, HBS, MMS)
- Lager beroeps Onderwijs (LBO/VBO, LEAO, ambacht-, technische of
- huishoudschool of een interne bedrijfsopleiding) en MBO-kort
- Middelbaar Beroeps Onderwijs (MTS, MEAO, MBO-lang, BOL, BBL)
- Hoger Beroeps Onderwijs (HTS, HEAO, MO-A)/ Universiteit
- Anders, namelijk ……………………………………

**5. Wat is uw beroep?**

………………………………………

**6. In welk land bent u geboren?**

……………………………………..

**7. In welk land zijn uw ouders geboren?**

Vader:………………………………….

Moeder: ……………………………….

**8. Bent u weleens in een boerderij geweest met Landbouwhuisdieren?**

**(bijvoorbeeld varkens, kippen, schapen, runderen/koeien, paarden, geiten)**

- Nee
- Ja, soms
- Ja, vaak

**8b. Heeft u direct contact (aanraking) gehad met de genoemde dieren?**

- **Nee**
- **Ja**
- Weet ik niet

**9 Heeft u ooit geelzucht gehad?**

(geelzucht wil zeggen: gele ogen en/of plotse gele huidskleur)

- Nee (ga naar vraag **10**)
- Ja
- Weet ik niet

**9b Hoelang geleden heeft u geelzucht gehad?**

- **…….. Jaar**
- **Onbekend**

1. **Heeft u in het verleden een bloedtransfusie ondergaan (donorbloed ontvangen)?**

- Nee
- Ja

Zo ja, in welk(e) land(en)? Land……………. Jaar………………..

Land…………….. Jaar…………………

Land……………… Jaar………………….

- Weet ik niet

1. **Bent u ooit een periode van 3 maanden of langer in een land buiten West-Europa, Noord-Amerika( Verenigde Staten), Nieuw-Zeeland en Australië geweest?**

- Nee
- Ja

Zo ja, in welk(e) continent(en) bent u geweest?

- Azië Jaar………………..
- Afrika Jaar…………………
- Midden/zuid Amerika Jaar………………….

**De volgende vragen gaan over uw eetgewoonten sinds uw 12^e^ jaar of de afgelopen 10 jaar**

1. **Volgt u een bepaalde leefregel bij het eten?**

- Nee
- Ja

**Zo ja, welke leefregel** **volgt u?** (meer antwoorden mogelijk)

- Vegetarisch (geen vlees/kip), maar **wel** vis of schaaldieren (ga naar vraag **22**)
- Vegetarisch (geen vlees/kip) én **geen** vis of schaaldieren **--> Einde van de vragenlijst**
- Veganistisch: helemaal geen dierlijke producten**--> Einde van de vragenlijst**
- Religieuze leefregels, zoals Halal of Kosher
- Anders, namelijk,………………………………………………………………………………

1. **Eet u vleeswaren?**

- Nee (ga naar vraag **14**)
- Ja

Zo ja, hoe vaak gemiddeld:

| Minder dan 1 dag per 4 weken | 1 dag  per 4 weken | 2-3 dgn  per 4  weken | 1 dag  per  week |
| --- | --- | --- | --- |
| □ | **□** | **□** | **□** |

**13b Welke soorten vleeswaren eet u?**  (Omcirkel de vleeswaren die bij u van toepassing zijn)

Filet américain, (smeer)leverworst, leverpastei, paté, berliner/leverkaas, hausmacher, gekookte lever, ham, kipfilet, kiprollade, rookvlees, rosbief, fricandeau, casselerib, ossenworst, chorizo, cervelaatworst, snijworst, metworst, salami, ontbijtspek, katenspek, bacon, luncheon meat, Boterhamworst, corned beef, gebraden gehakt, gekookte worst, palingworst, knakworst, zure zult, carpaccio.

1. **Eet u orgaanvlees, zoals lever, niertjes, hartjes etc. bij de broodmaaltijd, warme maaltijd of als tussendoortje?**

- Nee ( ga naar vraag **15**)
- Ja

Zo ja, hoe vaak gemiddeld:

| Minder dan 1 dag per 4 weken | 1 dag  per 4 weken | 2-3 dgn  per 4  weken | 1 dag  per  week |
| --- | --- | --- | --- |
| □ | **□** | **□** | **□** |

**14b Welke soorten orgaanvlees eet u meestal?** (meerdere antwoorden mogelijk)

□ Lever

□ Ander orgaanvlees, zoals niertjes, hartjes etc.

**14c Eet u weleens rauw of half gaar orgaanvlees?**

- Nee
- Ja
- Weet ik niet

1. **Eet u kip bij de broodmaaltijd, warme maaltijd of als tussendoortje?**

- Nee
- Ja

Zo ja, hoe vaak:

| Minder dan 1 dag per 4 weken | 1 dag  per 4 weken | 2-3 dgn  per 4  weken | 1 dag  per  week |
| --- | --- | --- | --- |
| □ | **□** | **□** | **□** |

1. **Eet u gehakt bij de broodmaaltijd, warme maaltijd of als tussendoortje?**

- Nee (ga naar vraag **17**)
- Ja

Zo ja, hoe vaak gemiddeld:

| Minder dan 1 dag per 4 weken | 1 dag  per 4 weken | 2-3 dag  per 4  weken | 1 dag  per  week |
| --- | --- | --- | --- |
| □ | **□** | **□** | **□** |

**16b Welke soorten gehakt eet u meestal?** (meerdere antwoorden mogelijk)

□ Rundergehakt

□ Half-om-half gehakt

□ Andere soorten gehakt

**16c Eet u weleens rauw of half gaar gehakt?**

- Nee
- Ja
- Weet ik niet

1. **Eet u rundvlees bij de broodmaaltijd, warme maaltijd of als tussendoortje?**

**Let op:** gehakt niet meetellen

- Nee (ga naar vraag **18**)
- Ja

Zo ja, hoe vaak:

| Minder dan 1 dag per 4 weken | 1 dag  per 4 weken | 2-3 dgn  per 4  weken | 1 dag  per  week |
| --- | --- | --- | --- |
| □ | **□** | **□** | **□** |

**17b** **Welke soorten rundvlees eet u?** (Omcirkel de vleessoorten die bij u van toepassing zijn)

Biefstuk, rosbief, tartaar, poulet, magere runderlappen, doorregen runderlappen, entrecote, riblappen, sukadelappen, runderbraadworst, ander rundvlees

**17c Eet u weleens rauw of half gaar rundvlees?**

- Nee
- Ja
- Weet ik niet

**18. Eet u varkensvlees bij de broodmaaltijd, warme maaltijd of als tussendoortje?**

**Let op:** gehakt niet meetellen

- Nee ( ga naar vraag **19**)
- Ja

Zo ja, hoe vaak:

| Minder dan 1 dag per 4 weken | 1 dag  per 4 weken | 2-3 dgn  per 4  weken | 1 dag  per  week |
| --- | --- | --- | --- |
| □ | **□** | **□** | **□** |

**18b** **Welke soorten varkensvlees eet u?** (Omcirkel de vleessoorten die bij u van toepassing zijn)

Hamlap, schnitzel, fricandeau, varkenshaas, karbonade, filetlap, hamschijf, schouderlap, varkensshoarmavlees, speklap, spekjes, slavink, saucijsjes/braadworst, spareribs, ander varkensvlees

**18c Eet u weleens rauw of half gaar orgaanvlees?**

- Nee
- Ja
- Weet ik niet

1. **Eet u rookworst of knakworst bij de broodmaaltijd, warme maaltijd of als tussendoortje?**

- Nee
- Ja

Zo ja, hoe vaak:

| Minder dan 1 dag per 4 weken | 1 dag  per 4 weken | 2-3 dag  per 4  weken | 1 dag  per  week |
| --- | --- | --- | --- |
| □ | **□** | **□** | **□** |

1. **Eet u ander vlees, zoals kalfs-, lams-, schapen- en/of geitenvlees, eend, konijn etc. bij de broodmaaltijd, warme maaltijd of als tussendoortje?**

- Nee (ga naar vraag **21**)
- Ja

Zo ja, hoe vaak:

| Minder dan 1 dag per 4 weken | 1 dag  per 4 weken | 2-3 dag  per 4  weken | 1 dag  per  week |
| --- | --- | --- | --- |
| □ | **□** | **□** | **□** |

**20b** **Eet u weleens rauw of half gaar andere vleessoorten?**

- Nee
- Ja
- Weet ik niet

1. **Eet u wild, zoals hert, haas, wilde zwijn etc. bij de broodmaaltijd, warme maaltijd of als tussendoortje?**

- Nee (ga naar vraag **22**)
- Ja

Zo ja, hoe vaak:

| Minder dan 1 dag per 4 weken | 1 dag  per 4 weken | 2-3 dag  per 4  weken | 1 dag  per  week |
| --- | --- | --- | --- |
| □ | **□** | **□** | **□** |

**21b Eet u weleens rauw of half gaar wild?**

- Nee
- Ja
- Weet ik niet

1. **Eet u mosselen, garnalen, krab, kreeft, inktvis of andere schaal- en schelpdieren?**

- Nee (ga naar vraag **23**)
- Ja

Zo ja, hoe vaak:

| Minder dan 1 dag per 4 weken | 1 dag  per 4 weken | 2-3 dag  per 4  weken | 1 dag  per  week |
| --- | --- | --- | --- |
| □ | **□** | **□** | **□** |

**22b** **Welke soorten schaal- en schelpdieren eet u meestal?** (meer antwoorden mogelijk)

- Mosselen
- Andere schaal- en schelpdieren, zoals garnalen, krab, kreeft, inktvis etc.

**22c Eet u weleens rauw of half gaar schaal- en schelpdieren?**

- Nee
- Ja
- Weet ik niet

1. **Eet u vis bij uw broodmaaltijd, warme maaltijd of als tussendoortje?**

**Let op:** Schaal- en schelpdieren niet meetellen.

- Nee
- Ja

Zo ja, hoe vaak:

| Minder dan 1 dag per 4 weken | 1 dag  per 4 weken | 2-3 dag  per 4  weken | 1 dag  per  week |
| --- | --- | --- | --- |
| □ | **□** | **□** | **□** |

**Dit is het einde van de vragenlijst. Hartelijk bedankt!**

# Hepatitis E virus food frequency questionnaire

In this questionnaire both general and specific questions concerning risk factors for an infection with hepatitis E virus infection are asked. This questionnaire will be analysed anonymously. This means that your answers will be handled in the strictest confidence and cannot be traced back to you personally.

Completing the questionnaire will take about 10 – 20 minutes.

We want to thank you for your cooperation!

**Date** …./…./….……

**Since you were 12 years old, or In the past 10 years, how often did you eat meat, fish or crustaceans?**

- Never
- 1 time a week or less
- More often **🡪** **End of questionnaire: you cannot participate in this study**

Sticker

**1. What are the 4 numbers of your postal code?** 🞎🞎🞎🞎

**2. Are you a man or a woman?**

- Man
- Woman

**3. What is your date of birth?**

day month year

4**. What is the highest level of education that you have had or are presently following?**

- No school education
- Primary school
- Lower general secondary education (MAVO, MULO, ULO)
- Higher general secondary education (HAVO, VWO, HBS, MMS)
- Lower vocational education (LBO/VBO, LEAO, craft-, technical or domestic school or an internal company training) and short track MBO
- Secondary vocational education (MTS, MEAO, long track-MBO, BOL, BBL)
- Polytechnic (HTS, HEAO, MO-A)/ University
- Other: ……………………………………

**5. What is your profession?**

………………………………………

**6. What is your country of birth?**

……………………………………..

**7. In which country were your parents born?**

Father:………………………………….

Mother: ……………………………….

**8 Did you ever visit a farm with farm animals?**

**(for example pigs, chicken, sheep, cows, horses, goats)**

- No
- Yes, sometimes
- Yes, often

**8b. Did you have direct contact (by touching) with any of these animals?**

- No
- Yes
- Do not know

**9 Have you ever had jaundice?**

(jaundice means: yellow eyes and/or a sudden yellowish skin colour)

- No (go to question 10)
- Yes
- Do not know

**9b How long ago did you have jaundice?**

- ……..Year(s)
- Do not know

1. **Did you ever in the past receive a blood transfusion (receive donor blood)?**

- No
- Yes

If yes, in which country/ countries?
 Country……………. Year………………..

Country…………….. Year…………………

Country……………… Year………………….

- Do not know

1. **Have you ever visited a country outside of Western Europe, North America (United States) New Zealand or Australia for a period of 3 months or longer?**

- No
- Yes

If yes, in which continents have you been?

- Asia Year………………..
- Africa Year…………………
- Middle/South America Year………………….

**The next series of questions concerns your eating habits since you were 12 years old, or during the past 10 years.**

1. **Do you follow a specific lifestyle concerning your eating behaviour?**

- No
- Yes

**If yes, which style?** (several answers may apply)

- Vegetarian (no meat/chicken), but I **do eat** fish or shellfish (go to question 22)
- Vegetarian (no meat/chicken) and **also** **no** fish or shellfish **--> end of the questionnaire**
- Vegan: no animal products at all**--> end of the questionnaire**
- Religious lifestyle such as Halal or Kosher
- Other:,………………………………………………………………………………

1. **Do you eat meat products on bread?**

- No (go to question **14**)
- Yes

If yes, how often on average:

| Less than 1 day per 4 weeks | 1 day  per 4 weeks | 2-3 days  per 4  weeks | 1 day  per  week |
| --- | --- | --- | --- |
| □ | **□** | **□** | **□** |

**13b Which types of meat products do you eat?**  (Circle all that apply)

Filet Américain, (spreadable) liver sausage, liver paste, pâté, Berliner/liver cheese, Hausmacher, cooked liver, ham, chicken breast, chicken roll, smoked meat, roast beef, fricandeau, Kassler, ox sausage, chorizo, cervelat sausage, cut sausage, metworst (dry Dutch sausage made of raw minced pork), salami, bacon, luncheon meat, corned beef, fried minced meat, cooked sausage, ‘eel’ sausage, frankfurters, sour sausage, Carpaccio.

1. **Do you eat organ meat, such as liver, kidney, heart, etc. at breakfast, lunch or dinner or in between meals?**

- No (go to question **15**)
- Yes
  If yes, how often on average:

| Less than 1 day per 4 weeks | 1 day  per 4 weeks | 2-3 days  per 4  weeks | 1 day  per  week |
| --- | --- | --- | --- |
| □ | **□** | **□** | **□** |

**14b Which type of organ meat do you eat most?** (several answers may apply)

□ Liver

□ Other organ meat such as kidney, heart, etc.

**14c Do you ever eat raw or undercooked organ meat?**

- No
- Yes
- Do not know

1. **Do you eat chicken at breakfast, lunch or dinner or in between meals?**

- No (go to question **16**)
- Yes

If yes, how often:

| Less than 1 day per 4 weeks | 1 day  per 4 weeks | 2-3 days  per 4  weeks | 1 day  per  week |
| --- | --- | --- | --- |
| □ | **□** | **□** | **□** |

1. **Do you eat minced meat at breakfast, lunch or dinner or in between meals?**

- No (go to question **17**)
- Yes

If yes, how often on average:

| Less than 1 day per 4 weeks | 1 day  per 4 weeks | 2-3 days  per 4  weeks | 1 day  per  week |
| --- | --- | --- | --- |
| □ | **□** | **□** | **□** |

**16b Which types of minced meat do you eat most often?** (several answers may apply)

□ Minced beef

□ Half beef-half pork minced meat

□ Other types of minced meat

**16c Do you ever eat raw or undercooked minced meat?**

- No
- Yes
- Do not know

1. **Do you eat beef at breakfast, lunch or dinner or in between meals?**

**Attention:** exclude minced beef

- No (go to question**18**)
- Yes

If yes, how often:

| Less than 1 day per 4 weeks | 1 day  per 4 weeks | 2-3 days  per 4  weeks | 1 day  per  week |
| --- | --- | --- | --- |
| □ | **□** | **□** | **□** |

**17b** **Which types of beef to you eat?** (Circle all that apply)

Steak, roast beef, tartar, poulet, skinny beef rags, marbled beef steak, entrecote, ribs, meat slices, beef bratwurst, other beef meat

**17c Do you ever eat raw or undercooked beef meat?**

- No
- Yes
- Do not know

1. **Do you eat pork at breakfast, lunch or dinner or in between meals?**

**Attention:** exclude minced meat

- No (go to question **19**)
- Yes

If yes, how often:

| Less than 1 day per 4 weeks | 1 day  per 4 weeks | 2-3 days  per 4  weeks | 1 day  per  week |
| --- | --- | --- | --- |
| □ | **□** | **□** | **□** |

**18b** **Which types of pork meat to you eat?** (Circle all that apply)

Ham, schnitzel, fricandeau, tenderloin, ‘carbonade’, filet, ham disk, shoulder patch, pork Shawarma, bacon, ‘slavink’ (minced meat wrapped in bacon), sausages/bratwurst, spareribs, other pork meat

**18c Do you ever eat raw or undercooked pork meat?**

- No
- Yes
- Do not know

1. **Do you eat smoked sausage or frankfurter (cooked sausage that are scalded) at breakfast, lunch or dinner or in between meals?**

- No
- Yes

If yes, how often:

| Less than 1 day per 4 weeks | 1 day  per 4 weeks | 2-3 days  per 4  weeks | 1 day  per  week |
| --- | --- | --- | --- |
| □ | **□** | **□** | **□** |

1. **Do you eat other meat types such as veal, lamb, sheep, goat, duck, rabbit, etc. at breakfast, lunch or dinner or in between meals?**

- No (go to question **21**)
- Yes

If yes, how often:

| Less than 1 day per 4 weeks | 1 day  per 4 weeks | 2-3 days  per 4  weeks | 1 day  per  week |
| --- | --- | --- | --- |
| □ | **□** | **□** | **□** |

**20b** **Do you ever eat raw or undercooked other meat types?**

- No
- Yes
- Do not know

1. **Do you eat wild meat such as deer, hare, wild boar, etc. at breakfast, lunch or dinner or in between meals?**

- No (go to question **22**)
- Yes

If yes, how often:

| Less than 1 day per 4 weeks | 1 day  per 4 weeks | 2-3 days  per 4  weeks | 1 day  per  week |
| --- | --- | --- | --- |
| □ | **□** | **□** | **□** |

**21b Do you ever eat raw or undercooked wild meat?**

- No
- Yes
- Do not know

1. **Do you eat mussels, shrimps, crab, lobster, squid or other shellfish?**

- No (go to question **23**)
- Yes

If yes, how often:

| Less than 1 day per 4 weeks | 1 day  per 4 weeks | 2-3 days  per 4  weeks | 1 day  per  week |
| --- | --- | --- | --- |
| □ | **□** | **□** | **□** |

**22b** **Which type of shellfish or crustaceans do you eat most often?** (several answers may apply)

- Mussels
- Other shellfish such as shrimps, crab, lobster, squid etc..

**22c Do you ever eat raw or undercooked shellfish or crustaceans?**

- No
- Yes
- Do not know

1. **Do you eat fish at breakfast, lunch or dinner or in between meals?
   Attention:** Exclude shellfish and crustacean.

- No
- Yes

If yes, how often:

| Less than 1 day per 4 weeks | 1 day  per 4 weeks | 2-3 days  per 4  weeks | 1 day  per  week |
| --- | --- | --- | --- |
| □ | **□** | **□** | **□** |

**This is the end of the questionnaire. Thank you very much for your cooperation!**
