# Supplementary material for: Hepatitis E virus seroprevalence and determinants in various study populations in the Netherlands
Source: PLoS One. 2018 Dec 17;13(12):e0208522. doi: 10.1371/journal.pone.0208522 (PMC6296558; doi:10.1371/journal.pone.0208522)
Supplement: S3 File — (DOCX) [file pone.0208522.s004.docx]

**Anoniem onderzoek naar het vóórkomen van seksueel- en bloedoverdraagbare aandoeningen onder bezoekers van de SOA–polikliniek van de GGD Amsterdam**

Graag willen wij alle bezoekers van 16 jaar en ouder vragen om mee te werken aan een onderzoek naar het vóórkomen van en mogelijke risicofactoren voor een infectie met seksueel- en bloedoverdraagbare aandoeningen, waaronder HIV. Lees onderstaande informatie goed door om te kunnen beslissen of u hieraan wilt meewerken.

**Doel**

Door middel van dit onderzoek wil de GGD een beter inzicht krijgen in het vóórkomen van seksueel- en bloedoverdraagbare aandoeningen, waaronder HIV-infecties, bij bezoekers van de SOA-polikliniek.

**Wat houdt het onderzoek in?**

Deelname aan het onderzoek houdt in dat wij u enkele vragen stellen en dat, tegelijk met de bloedafname voor het reguliere SOA-onderzoek (op syfilis en eventueel HIV), twee extra buisjes bloed worden afgenomen voor onderzoek naar seksueel- en bloedoverdraagbare aandoeningen zoals antistoffen tegen het HIV-virus. Het is dus niet nodig dat u hiervoor opnieuw geprikt moet worden. Er zijn geen extra risico’s verbonden aan het afnemen van het bloed; dit heeft ook geen nadelige invloed op uw conditie of gezondheid.

Uw antwoorden op de vragenlijst en gegevens waaronder bijvoorbeeld uw leeftijd en de uitslagen van het reguliere SOA-onderzoek zullen later worden toegevoegd aan de uitslagen van deze studie, om deze studieuitslagen beter te kunnen begrijpen.

De antwoorden op de vragen en de resultaten van het bloedonderzoek, naar onder andere HIV, zijn niet te herleiden tot uw persoon. Het is daarom niet mogelijk om u de uitslag van dit bloedonderzoek mee te delen. Als u een HIV-test met uitslag wilt, kunt u dit bespreken met de verpleegkundige die u onderzoekt. Er zal dan ook een HIV-test gedaan worden als onderdeel van het standaard SOA-onderzoek.

**Vrijwillige deelname**

Uw deelname aan dit onderzoek is geheel vrijwillig. Wanneer u besluit niet meer mee te willen doen met dit onderzoek heeft dat geen gevolgen voor het reguliere SOA-onderzoek en de eventuele behandeling van geslachtsziekten. Het is echter van groot belang dat zoveel mogelijk bezoekers van de polikliniek aan dit onderzoek meewerken. Uw medewerking wordt dan ook zeer op prijs gesteld.

**Toestemming**

Bij deelname aan het onderzoek gaat u er mee akkoord dat er bloed en vragenlijsten worden afgenomen, en dat dit voor onbepaalde tijd wordt opgeslagen voor wetenschappelijk onderzoek op het gebied van seksueel- en bloedoverdraagbare aandoeningen, waaronder HIV.

**Verzekering**

Aangezien aan deelname aan deze studie geen risico’s verbonden zijn, heeft de Medisch Ethische Commissie van het AMC, die dit onderzoek beoordeeld heeft, ontheffing verleend van de verplichting voor de deelnemers een speciale schadeverzekering af te sluiten.

**Nadere informatie**

Voor meer informatie over dit onderzoek kunt u terecht bij de verpleegkundige of arts die u onderzoekt. Ook kunt u een onafhankelijk arts raadplegen voor vragen. Voor dit onderzoek is dat de heer XXXX, arts op de afdeling Reizigersvaccinatie en Infectieziekten, GGD Amsterdam, telefoonnummer: 020- XXX XXX.

Bedankt voor uw medewerking!

**Vragenlijst studie populatie D**

Datum: …../…../…..(dd/mm/jjjj) *(automatisch datum van vandaag)*

Oproepnummer

Naam interviewer:………. …….

Is de cliënt een deelnemer of weigeraar?

- Deelnemer *Ga naar vraag 1*
- Weigeraar

Reden weigering:

- Participatie
- Geen extra bloed
- Geen tijd
- Niet meewerken wetenschappelijk onderzoek
- Geen persoonlijk belang
- Onderzoek te confronterend
- Taalbarrière
- Overige, nl………………

1. Geboortejaar 19

2. Wat is uw geslacht?

- Man
- Vrouw

3. Hoeveel seksuele partners heeft u in de afgelopen 6 maanden gehad?

…….mannen

…….vrouwen

**Vragenlijst studie populatie E**

Datum: …../…../…..(dd/mm/jjjj) *(automatisch datum van vandaag)*

Oproepnummer

Naam interviewer:………. …….

Is de cliënt een deelnemer of weigeraar?

- Deelnemer *Ga naar vraag 1*
- Weigeraar

Reden weigering:

- Participatie
- Geen extra bloed
- Geen tijd
- Niet meewerken wetenschappelijk onderzoek
- Geen persoonlijk belang
- Onderzoek te confronterend
- Taalbarrière
- Overige, nl………………

1. Geboortejaar 19

2. Wat is uw geslacht?

- Man
- Vrouw

3. Heeft u in het afgelopen half jaar gespoten?

- Nee
- Ja

4. Heeft u ooit gespoten?

- Nee
- Ja

**Anonymous study of the presence of sexually- and blood transmitted infections among visitors of the Sexually Transmitted Infections (STI) clinic of the Health Service (GGD) of Amsterdam**

We would like to ask all visitors of the STI clinic, aged 16 years and older, to participate in a study for the presence of, and the possible risk factors for, acquiring sexually- or blood transmitted infections, including HIV. Please read the infomation below carefully, so you can decide if you want to participate.

**Aim of the study**

With this study, the Municipal Health Service of Amsterdam wants to gain insight into the presence of sexually- and blood transmitted infections, including HIV-infections, among visitors of the STI clinic.

**What does participation mean?**

Participating in this study means that we will ask you some questions, and that, simultaneously with the withdrawal of blood for the routine STI screening (for syphilis and possibly HIV), we will withdraw two extra tubes of blood for the anonymous screening for sexually- and blood transmitted infections, such as the screening for antibodies for the HIV-virus. Therefore, it is unnecessary for the attending nurse withdraw blood twice. There are no extra risks involved in the withdrawal of blood; moreover, it does not have a negative influence on your physical condition or your health.

Your responses to the questions, and other information, such as your age, and the results of the routine STI screening, will be added to the results of this study at a later stage, to gain a better understanding of these study results.

The answers to the questions and the results of the anonymous blood test for this study, including HIV, cannot be traced back to you personally. As a consequence, it is impossible to inform you of the results of this blood test. If you want to do an HIV-test and be informed of its result, please discuss this with the attending nurse. The HIV-test will then be done as part of the routine STI screening.

**Voluntary participation**

Your participation in this study is completely voluntary. If you decide not to participate or to withdraw your permission, this has no consequences for the routine STI screening or possible treatment for an STI. But, it is of the utmost importance that as many visitors as possible participate in this study, so your cooperation and participation is much appreciated!

**Permission**

By participating in this study, you agree that your blood will be drawn and you agree with filling out a questionnaire. Furthermore, you agreethat your blood and your answers to the questions wil be stored for an unspecified period of time for scientific research on sexually- and blood transmitted infections, including HIV.

**Insurance**

The Ethical Review Board of the Academical Medical Center (AMC), which has reviewed this study, has granted participants dispensation from the obligation to have a special indemnity insurance, since participation in this study involves no risks.

**Further information**

For more information about this study, you can approach the attending nurse or doctor. You can also consult an independent doctor for any questions you may have. For this study, the independent doctor is dr. XXX, physician at the department of Vaccinations and Infectious Diseases, Municipal Health Service of Amsterdam, telephone number: 020-XXX XXX.

**Thank you for your cooperation!**

**Questionnaire study population D**

Datum: …../…../…..(dd/mm/jjjj) *(automatisch datum van vandaag)*

Oproepnummer

Naam interviewer:………. …….

Does the visitor wants to participate?

- Participates *Ga naar vraag 1*
- Refuses

Reason for refusal:

- No extra blood
- No time
- Does not want to cooperate in scientific research
- No personal interest
- Survey too confronting (questions)
- Language issue
- Other, namely………………

1.Year of birth 19

2. What is your gender?

- Male
- Female

3. How many sexual partners did you have in the preceding six months?

…….male partners (number)

…….female partners

**Questionnaire study population E**

Datum: …../…../…..(dd/mm/jjjj) *(automatisch datum van vandaag)*

Oproepnummer

Naam interviewer:………. …….

Does the visitor wants to participate?

- Participates *Ga naar vraag 1*
- Refuses

Reason for refusal:

- No extra blood
- No time
- Does not want to cooperate in scientific research
- No personal interest
- Survey too confronting (questions)
- Language issue
- Other, namely………………

1.Year of birth 19

2. What is your gender?

- Male
- Female

3. Did you inject drugs in the preceding six months?

- Nee
- Ja

4. Did you ever inject drugs in the preceding six months?

- Nee
- Ja
